# Supplementary material for: Literary Identification of Differentially Hydroxymethylated DNA Regions for Type 2 Diabetes Mellitus: A Scoping Minireview
Source: Int J Environ Res Public Health. 2024 Feb 4;21(2):177. doi: 10.3390/ijerph21020177 (PMC10887687; doi:10.3390/ijerph21020177)
Supplement: Supplementary file 1 [file ijerph-21-00177-s001.zip › ijerph-2835072-supplementary.pdf]

Supplementary Materials

Table S1. Search syntaxes

<table>
<tr><th>Search</th><th colspan="2">Syntax</th><th>Results</th></tr>
<tr><td rowspan="2">#1</td><td colspan="2">5-hydroxymethylcytosine" [Supplementary Concept]) AND "Diabetes Mellitus, Type 2"[Mesh]</td><td rowspan="2">3</td></tr>
<tr><td colspan="2">("5-Methylcytosine/analogs and derivatives"[Mesh]) AND "Diabetes Mellitus, Type 2"[Mesh]</td></tr>
<tr><td rowspan="21">#2</td><td>Search no.</td><td>PubMed Keyword search strategy</td><td rowspan="21">12</td></tr>
<tr><td>#1</td><td>“5hmC & T2DM”</td></tr>
<tr><td>#2</td><td>“5hmC & Type 2 Diabetes”</td></tr>
<tr><td>#3</td><td>“DNA Hydroxymethylation & Type 2 Diabetes”</td></tr>
<tr><td>#4</td><td>“5hmC, human, T2DM”</td></tr>
<tr><td>#5</td><td>“hydroxymethylation, human, T2DM”</td></tr>
<tr><td>#6</td><td>“5hmC, human, T2DM”</td></tr>
<tr><td>#7</td><td>“5hmC, patient, T2DM”</td></tr>
<tr><td>#8</td><td>“5-hydroxymethylcytosine, human, T2DM”</td></tr>
<tr><td>#9</td><td>“5hmC, people, T2DM”</td></tr>
<tr><td>#10</td><td>“hydroxymethylation, people, T2DM”</td></tr>
<tr><td>#11</td><td>“hydroxymethylation, patients, T2DM”</td></tr>
<tr><td>#12</td><td>“DNA hydroxymethylation T2DM”</td></tr>
<tr><td>#13</td><td>“DNA hydroxymethylation type 2 diabetes”</td></tr>
<tr><td>#14</td><td>“5-hydroxymethylcytosine, type 2 diabetes”</td></tr>
<tr><td>#15</td><td>“5-hydroxymethylcytosine, type 2 diabetes, human”</td></tr>
<tr><td>#16</td><td>“5-hydroxymethylcytosine, type 2 diabetes, patients”</td></tr>
<tr><td>#17</td><td>“5hmC, T2DM”</td></tr>
<tr><td>#18</td><td>“5-hydroxymethylcytosine, T2DM”</td></tr>
<tr><td>#19</td><td>“5hmc, T2DM, Peripheral blood”</td></tr>
<tr><td>#20</td><td>“5-hydroxymethylcytosine, T2DM, peripheral blood”</td></tr>
</table>
